# Water‐, pH‐ and temperature relations of germination for the extreme xerophiles *Xeromyces bisporus* (FRR 0025), *Aspergillus penicillioides* (JH06THJ) and *Eurotium halophilicum* (FRR 2471)

**DOI:** 10.1111/1751-7915.12406

**Published:** 2016-08-26

**Authors:** Andrew Stevenson, Philip G. Hamill, Jan Dijksterhuis, John E. Hallsworth

**Affiliations:** ^1^ Institute for Global Food SecuritySchool of Biological SciencesMBCQueen's University BelfastBelfastBT9 7BLUK; ^2^ CBS‐KNAW Fungal Biodiversity CentreUppsalalaan 8CT 3584UtrechtThe Netherlands

## Abstract

Water activity, temperature and pH are determinants for biotic activity of cellular systems, biosphere function and, indeed, for all life processes. This study was carried out at high concentrations of glycerol, which concurrently reduces water activity and acts as a stress protectant, to characterize the biophysical capabilities of the most extremely xerophilic organisms known. These were the fungal xerophiles: *Xeromyces bisporus* (FRR 0025), *Aspergillus penicillioides* (JH06THJ) and *Eurotium halophilicum* (FRR 2471). High‐glycerol spores were produced and germination was determined using 38 media in the 0.995–0.637 water activity range, 33 media in the 2.80–9.80 pH range and 10 incubation temperatures, from 2 to 50°C. Water activity was modified by supplementing media with glycerol+sucrose, glycerol+NaCl and glycerol+NaCl+sucrose which are known to be biologically permissive for *X. bisporus*,* A. penicillioides* and *E. halophilicum* respectively. The windows and rates for spore germination were quantified for water activity, pH and temperature; symmetry/asymmetry of the germination profiles were then determined in relation to *supra*‐ and sub‐optimal conditions; and pH‐ and temperature optima for extreme xerophilicity were quantified. The windows for spore germination were ~1 to 0.637 water activity, pH 2.80–9.80 and > 10 and < 44°C, depending on strain. Germination profiles in relation to water activity and temperature were asymmetrical because conditions known to entropically disorder cellular macromolecules, i.e. *supra*‐optimal water activity and high temperatures, were severely inhibitory. Implications of these processes were considered in relation to the *in‐situ* ecology of extreme conditions and environments; the study also raises a number of unanswered questions which suggest the need for new lines of experimentation.

## Introduction

Extremely xerophilic fungi act as pioneers under hostile conditions, and can catalyse ecosystem development at low water‐availability. For instance, they can drive saprotrophic processes in arid soils, salt‐saturated brines and other water‐constrained environments such as stone surfaces, paper and other artefacts and high‐solute foodstuffs. Understanding xerophile behaviour in relation to environmental constraints also facilitates our understanding of the biophysical limits for life and addresses important questions relating to the issue of habitability of hostile environments (e.g. saline and high‐sugar habitats; Lievens *et al*., 2015; Stevenson *et al*., 2015a,b), including those which lie at the heart of the astrobiology field (Rummel *et al*., 2014).

Over the past 100‐year period, there have been occasional reports of germination and/or mycelial growth of extreme xerophiles at extremely low water‐activity (≥ 0.710) (Stevenson *et al*., 2015a,b). The majority of these provide single data‐points at which researchers have observed important processes, such as spore germination (e.g. Pitt and Christian, 1968). Fungal germination is not only a seminal moment in the fungal life‐cycle but it also facilitates dispersal and enables colonization of new substrates and habitats; it can enable development of new colonies in isolation from other microbes and/or bring fungi into contact with other living systems. Germination also represents a key biophysical event, whereby life exits dormancy to recover from a period of hostile conditions, such as prolonged desiccation or exposure to extremes of chaotropicity or temperature (Hallsworth *et al*., 2003a; Wyatt *et al*., 2015a,b; Yakimov *et al*., 2015).

A series of studies has been carried out to systematically identify the most‐xerophilic fungal genera and strains known to science (Williams and Hallsworth, 2009; Stevenson and Hallsworth, 2014; Stevenson *et al*., 2015a,b, in press). For the three most‐xerophilic genera of fungi, the strains able to grow and/or germinate at the lowest water activities are *Xeromyces bisporus* (FRR 0025), *Aspergillus penicillioides* (JH06THJ) and *Eurotium halophilicum* (FRR 2471). In the current study, these strains were used as models for the biophysical fringe of Earth's biosphere. The specific aims were to: (i) determine windows of water activity, pH and temperature which are permissive for spore germination; (ii) quantify rates of germination and germ‐tube extension in relation to these parameters; (iii) characterize the symmetry and/or asymmetry of the germination profiles in relation to *supra*‐ and sub‐optimal conditions; (iv) determine the pH‐ and temperature optima for xerophilicity (i.e. those values which facilitate germination at the lowest water‐activities); and (v) consider the implications of these germination processes for the *in‐situ* ecology of extreme conditions and environments. We put forward key, unanswered questions which suggest the need for new lines of experimentation.

## Results and discussion

### Biotic windows for germination

Spores were produced in high‐glycerol media (5.5 M glycerol, 0.821 water activity) and contained up to 15% w/v glycerol (Stevenson *et al*., in press). Strains produced D‐shaped ascospores (*X. bisporus*), conidia (*A. penicillioides*) or a mixture of ascospores and conidia (*E. halophilicum*). For *E. halophilicum*, germination was assessed for conidia only. The most‐permissive medium for germination of each xerophile strain (i.e. that which enables germination at low water‐activity) are glycerol+sucrose, glycerol+NaCl and glycerol+NaCl+sucrose for *X. bisporus* (FRR 0025), *A. penicillioides* (JH06THJ) and *E. halophilicum* (FRR 2471) respectively (Stevenson *et al*., in press); these media were used as the basis for the current study (Tables 1, 2, 3).

**Table 1:** Culture media used for germination assays of *Xeromyces bisporus* FRR 0025, *Aspergillus penicillioides* JH06THJ and *Eurotium halophilicum* FRR 2471 to characterize germination performance over the entire water‐activity window (see Fig. 1)^a^

Water activity^b^	Stressor type and concentration (M)^a^	pH
*Xeromyces bisporus* FRR 0025		
0.995	None	7.20
0.972	Glycerol (0.50)+sucrose (0.10)	7.20
0.920	Glycerol (1.70)+sucrose (0.20)	7.00
0.884	Glycerol (2.40)+sucrose (0.25)	6.90
0.849	Glycerol (3.00)+sucrose (0.35)	6.90
0.799	Glycerol (3.60)+sucrose (0.35)	6.70
0.762	Glycerol (4.30)+sucrose (0.40)	6.50
0.723	Glycerol (5.00)+sucrose (0.45)	6.50
0.699	Glycerol (5.50)+sucrose (0.50)	6.50
0.682	Glycerol (5.50)+sucrose (0.60)	6.50
0.674	Glycerol (5.50)+sucrose (0.65)	6.50
0.637	Glycerol (5.50)+sucrose (0.80)	6.30
*Aspergillus penicillioides* JH06THJ		
0.995	None	7.20
0.949	Glycerol (1.00)+NaCl (0.20)	7.10
0.917	Glycerol (1.50)+NaCl (0.30)	7.10
0.880	Glycerol (2.00)+NaCl (0.40)	6.90
0.868	Glycerol (2.50)+NaCl (0.40)	6.90
0.841	Glycerol (3.00)+NaCl (0.50)	6.80
0.824	Glycerol (3.50)+NaCl (0.50)	6.80
0.802	Glycerol (4.00)+NaCl (0.60)	6.80
0.787	Glycerol (4.50)+NaCl (0.70)	6.80
0.764	Glycerol (5.00)+NaCl (0.80)	6.80
0.741	Glycerol (5.50)+NaCl (1.00)	6.80
0.709	Glycerol (5.50)+NaCl (1.50)	6.70
0.692	Glycerol (5.50)+NaCl (1.60)	6.80
0.668	Glycerol (5.50)+NaCl (1.70)	6.80
0.640	Glycerol (5.50)+NaCl (1.80)	6.70
*Eurotium halophilicum* FRR 2471		
0.995	None	7.20
0.961	Glycerol (0.80)+NaCl (0.10)+sucrose (0.10)	7.20
0.939	Glycerol (1.00)+NaCl (0.20)+sucrose (0.20)	7.20
0.900	Glycerol (2.00)+NaCl (0.20)+sucrose (0.20)	7.00
0.875	Glycerol (2.50)+NaCl (0.30)+sucrose (0.30)	6.80
0.839	Glycerol (3.00)+NaCl (0.30)+sucrose (0.30)	6.90
0.823	Glycerol (3.50)+NaCl (0.40)+sucrose (0.40)	6.70
0.805	Glycerol (4.00)+NaCl (0.40)+sucrose (0.40)	6.70
0.771	Glycerol (4.50)+NaCl (0.50)+sucrose (0.50)	6.70
0.738	Glycerol (5.00)+NaCl (0.50)+sucrose (0.50)	6.80
0.701	Glycerol (5.50)+NaCl (0.50)+sucrose (0.30)	6.70
0.685	Glycerol (5.50)+NaCl (0.50)+sucrose (0.50)	6.70
0.651	Glycerol (5.50)+NaCl (0.80)+sucrose (0.50)	6.60

a All media were based on MYPiA: 1% malt extract, 1% yeast extract, 0.1% KH_2_PO_4_ and 1.5% (w/v) agar (Williams and Hallsworth, 2009).

b The water activity of each medium was measured at the temperature at which plates were incubated (30°C) and replicate values were within ± 0.002 units for the 1 to 0.900 water‐activity range and ± 0.001 units for the 0.900 to 0.600 water‐activity range (see *Experimental procedures*).

**Table 2:** Culture media used for germination assays of *Xeromyces bisporus* FRR 0025, *Aspergillus penicillioides* JH06THJ and *Eurotium halophilicum* FRR 2471 to characterize germination performance over the entire pH window (see Fig. 3)^a^

pH of culture medium^b^	Stressor type and concentration (M)^a^	Water activity^c^
*Xeromyces bisporus* FRR 0025		
2.90	Glycerol (5.5)+sucrose (0.3)	0.720
3.50	Glycerol (5.5)+sucrose (0.3)	0.719
4.60	Glycerol (5.5)+sucrose (0.3)	0.722
5.30	Glycerol (5.5)+sucrose (0.3)	0.721
5.80	Glycerol (5.5)+sucrose (0.3)	0.722
6.40	Glycerol (5.5)+sucrose (0.3)	0.722
7.10	Glycerol (5.5)+sucrose (0.3)	0.722
7.70	Glycerol (5.5)+sucrose (0.3)	0.719
8.10	Glycerol (5.5)+sucrose (0.3)	0.720
8.80	Glycerol (5.5)+sucrose (0.3)	0.718
9.60	Glycerol (5.5)+sucrose (0.3)	0.717
*Aspergillus penicillioides* JH06THJ		
3.00	Glycerol (5.5)+NaCl (1.2)	0.728
3.60	Glycerol (5.5)+NaCl (1.2)	0.725
4.70	Glycerol (5.5)+NaCl (1.2)	0.724
5.50	Glycerol (5.5)+NaCl (1.2)	0.727
6.00	Glycerol (5.5)+NaCl (1.2)	0.726
6.50	Glycerol (5.5)+NaCl (1.2)	0.729
7.00	Glycerol (5.5)+NaCl (1.2)	0.729
7.70	Glycerol (5.5)+NaCl (1.2)	0.724
8.40	Glycerol (5.5)+NaCl (1.2)	0.722
9.00	Glycerol (5.5)+NaCl (1.2)	0.726
9.80	Glycerol (5.5)+NaCl (1.2)	0.723
*Eurotium halophilicum* FRR 2471		
2.80	Glycerol (5.5)+NaCl (0.25)+sucrose (0.25)	0.723
3.70	Glycerol (5.5)+NaCl (0.25)+sucrose (0.25)	0.721
4.50	Glycerol (5.5)+NaCl (0.25)+sucrose (0.25)	0.724
5.50	Glycerol (5.5)+NaCl (0.25)+sucrose (0.25)	0.723
6.00	Glycerol (5.5)+NaCl (0.25)+sucrose (0.25)	0.725
6.40	Glycerol (5.5)+NaCl (0.25)+sucrose (0.25)	0.725
7.00	Glycerol (5.5)+NaCl (0.25)+sucrose (0.25)	0.726
7.50	Glycerol (5.5)+NaCl (0.25)+sucrose (0.25)	0.724
8.20	Glycerol (5.5)+NaCl (0.25)+sucrose (0.25)	0.721
8.90	Glycerol (5.5)+NaCl (0.25)+sucrose (0.25)	0.724
9.50	Glycerol (5.5)+NaCl (0.25)+sucrose (0.25)	0.719

a All media were based on MYPiA: 1% malt extract, 1% yeast extract, 0.1% KH_2_PO_4_ and 1.5% (w/v) agar (Williams and Hallsworth, 2009).

b Media were buffered by addition of citric acid/Na_2_PO_4_ (3.00, 3.75, 4.75 and 5.50), PIPES/NaOH (6.0, 6.5 and 7.2) or HEPES/NaOH (7.8, 8.5, 9.2, 10.1) pre‐autoclave. The pH of liquid media was measured using a Mettler Toledo Seven Easy pH‐probe (Mettler Toledo, Greifensee, Switzerland) and for solid media, post‐autoclave using Fisherbrand colour‐fixed pH indicator strips were used (Fisher Scientific, Leicestershire, UK).

c The water activity of each medium was measured at the same temperature at which plates were incubated (30°C) and replicate values were within ± 0.001 water activity (see *Experimental procedures*).

**Table 3:** Culture media used for germination assays of *Xeromyces bisporus* FRR 0025, *Aspergillus penicillioides* JH06THJ and *Eurotium halophilicum* FRR 2471 to characterize germination performance over the entire temperature window (see Fig. 4)^a^

Incubation temperature (°C)	Stressor type and concentration (M)^a^	Water activity^b^
*Xeromyces bisporus* FRR 0025		
2	Glycerol (5.5)+sucrose (0.3)	0.720
6	Glycerol (5.5)+sucrose (0.3)	0.719
10	Glycerol (5.5)+sucrose (0.3)	0.722
15	Glycerol (5.5)+sucrose (0.3)	0.721
20	Glycerol (5.5)+sucrose (0.3)	0.722
25	Glycerol (5.5)+sucrose (0.3)	0.722
30	Glycerol (5.5)+sucrose (0.3)	0.722
37	Glycerol (5.5)+sucrose (0.3)	0.719
44	Glycerol (5.5)+sucrose (0.3)	0.720
50	Glycerol (5.5)+sucrose (0.3)	0.718
*Aspergillus penicillioides* JH06THJ		
2	Glycerol (5.5)+NaCl (1.2)	0.728
6	Glycerol (5.5)+NaCl (1.2)	0.725
10	Glycerol (5.5)+NaCl (1.2)	0.724
15	Glycerol (5.5)+NaCl (1.2)	0.727
20	Glycerol (5.5)+NaCl (1.2)	0.726
25	Glycerol (5.5)+NaCl (1.2)	0.729
30	Glycerol (5.5)+NaCl (1.2)	0.729
37	Glycerol (5.5)+NaCl (1.2)	0.724
44	Glycerol (5.5)+NaCl (1.2)	0.722
50	Glycerol (5.5)+NaCl (1.2)	0.726
*Eurotium halophilicum* FRR 2471		
2	Glycerol (5.5)+NaCl (0.25)+sucrose (0.25)	0.723
6	Glycerol (5.5)+NaCl (0.25)+sucrose (0.25)	0.721
10	Glycerol (5.5)+NaCl (0.25)+sucrose (0.25)	0.724
15	Glycerol (5.5)+NaCl (0.25)+sucrose (0.25)	0.723
20	Glycerol (5.5)+NaCl (0.25)+sucrose (0.25)	0.725
25	Glycerol (5.5)+NaCl (0.25)+sucrose (0.25)	0.725
30	Glycerol (5.5)+NaCl (0.25)+sucrose (0.25)	0.726
37	Glycerol (5.5)+NaCl (0.25)+sucrose (0.25)	0.724
44	Glycerol (5.5)+NaCl (0.25)+sucrose (0.25)	0.721
50	Glycerol (5.5)+NaCl (0.25)+sucrose (0.25)	0.724

a All media were based on MYPiA: 1% malt extract, 1% yeast extract, 0.1% KH_2_PO_4_ and 1.5% (w/v) agar (Williams and Hallsworth, 2009).

b The water activity of each medium was measured at the same temperature at which plates were incubated and replicate values were within ± 0.001 water activity (see *Experimental procedures*).

**Figure 1:**
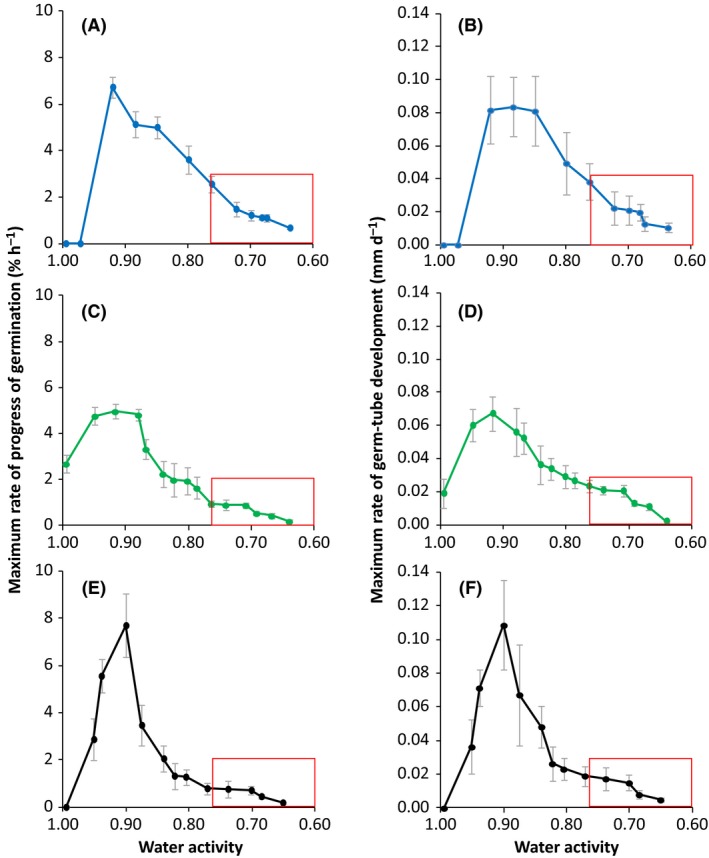
Maximum rates of spore germination (% of total h^−1^) and germ‐tube development for *Xeromyces bisporus* FRR 0025 (A and B), *Aspergillus penicillioides* JH06THJ (C and D) and *Eurotium halophilicum* FRR 2471 (E and F) over a range of water activity, on malt‐extract, yeast‐extract phosphate agar (MYPiA) supplemented with diverse stressor(s) and incubated at 30°C. For *X. bisporus*,* A. penicillioides* and *E. halophilicum*, media were supplemented with glycerol+sucrose, glycerol+NaCl or glycerol+NaCl+sucrose (respectively) over a range of concentrations to give a range of water activities from 0.995 to 0.637 (see Table 1). The red box indicates the water‐activity window selected for an additional study carried out to assess the potency of glycerol as a determinant for the water‐activity limit for life (*Experimental procedures*; Stevenson *et al*., in press). Maximum rates of germination and germ‐tube development were determined from the curves (data not shown) and grey bars indicate standard errors.

**Figure 2:**
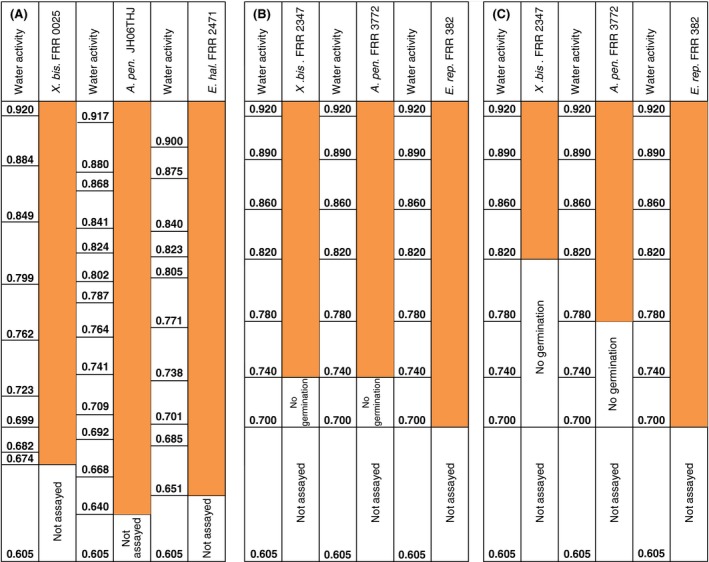
Comparisons of ability to germinate for diverse xerophile strains at sub‐optimal water‐activity values at 30°C by 30 days: (A) *X. bisporus* (FRR 0025), *A. penicillioides* (JH06THJ) and *E. halophilicum* (FRR 2471) (current study) and (B and C) *X. bisporus* (FRR 2347), *A. penicillioides* (FRR 3722) and *Eurotium repens* (FRR 382) (Gock *et al*., 2003); media were in the pH range 6.3–7.2 (A), and at pH 6.5 (B) and (C). Orange shading indicates germination within the 30‐day period.

To determine the windows of, and kinetics for, germination in relation to water activity,ranges of stressor concentrations were used for each strain (Table 1). The pH values of these media were close to neutral (i.e. in the range 6.3–7.2; Table 1) and plates were incubated at 30°C (see *Experimental procedures*). Collectively, the water activity windows for biotic activity of the xerophile strains (according to germination assays), as might be expected, spanned a range (0.995–0.637; Fig. 1) which was virtually equivalent to the water‐activity window for life on Earth (Stevenson *et al*., 2015a,b). There were, however, some slight differences between strains. Within the 30‐day time‐period of the study, *X. bisporus* (FRR 0025) did not germinate at 0.995 or 0.972 water activity, and *E. halophilicum* (FRR 2471) did not germinate at 0.995 water activity; by contrast, *A. penicillioides* (JH06THJ) was able to germinate on all media in the water‐activity range tested (0.995–0.640) (Fig. 1). An earlier study of xerophile germination reported limits of 0.820–0.740 for *X. bisporus* (FRR 2347), 0.780–0.740 for *A. penicillioides* (FRR 3772) and 0.700 for *Eurotium repens* (strain FRR 382), depending on pH, indicating that the strains used in the current study were considerably more xerophilic (Fig. 2; Gock *et al*., 2003). Furthermore, *X. bisporus* (FRR 0025) was not only able to germinate at 0.637 water activity, but it also did so at a reasonable rate implying that the actual water‐activity window is more extensive (Fig. 1A and B).

Earlier studies have suggested a higher water‐activity limit for mycelial extension than that observed for germination (Williams and Hallsworth, 2009). However, the current study indicates water‐activity minima for germination which are more or less equivalent to those reported for mycelial extension (Williams and Hallsworth, 2009; Stevenson *et al*., 2015a); possibly a consequence of the comparable types of (high‐glycerol) media used in these three studies. Without biotechnological interventions, many fungi have a water‐activity minimum for germination in the range 0.940–0.935 (Hallsworth and Magan, 1995; Hallsworth *et al*., 2003a,b; Lahouar *et al*., 2016). There are a few microbes known to have a water‐activity window for biotic activity that is comparable to that for the xerophiles in the current study (Stevenson *et al*., 2015a).

**Figure 3:**
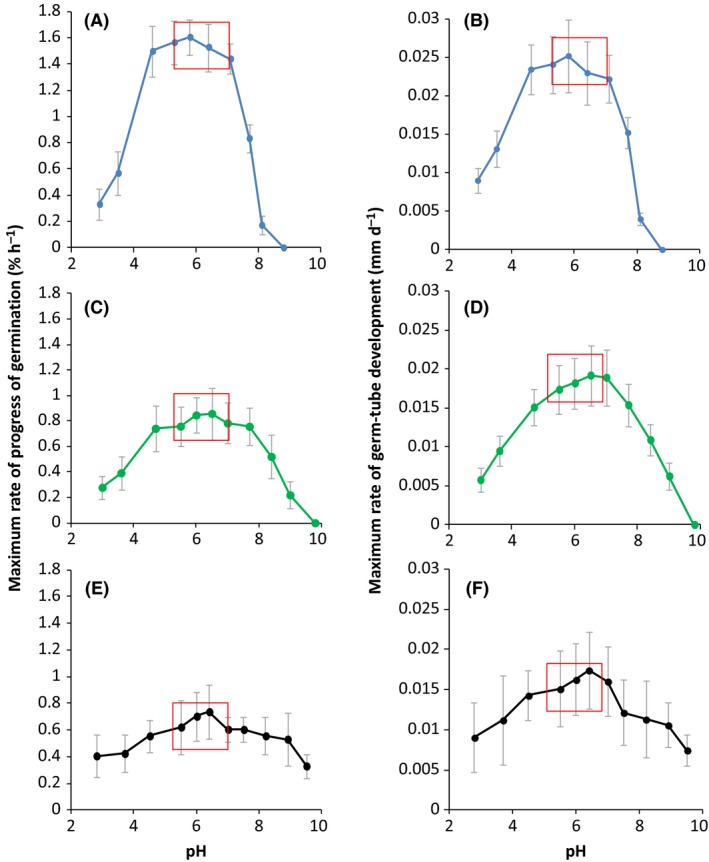
Maximum rates of spore germination (% of total h^−1^) and germ‐tube development for *Xeromyces bisporus* FRR 0025 (A and B), *Aspergillus penicillioides* JH06THJ (C and D) and *Eurotium halophilicum* (E and F) over a range of pH values, on malt‐extract, yeast‐extract phosphate agar (MYPiA) supplemented with diverse stressor(s), buffered and incubated at 30°C. For *X. bisporus*, media were supplemented with glycerol (5.5 M)+sucrose (0.4 M); for *A. penicillioides* with glycerol (5.5 M)+ NaCl (1.2 M) and for *E. halophilicum* with glycerol (5.5 M)+NaCl (0.25 M)+sucrose (0.25 M), and buffered to give pH values from 2.80 to 9.80 (see Table 2). The red box indicates the pH window selected for an additional study carried out to assess the potency of glycerol as a determinant for the water‐activity limit for life (*Experimental procedures*; Stevenson *et al*., in press). Maximum rates of germination and germ‐tube development were determined from the curves (data not shown) and grey bars indicate standard errors.

The pH windows for germination were considerable, spanning a range which was ~6,7 and 7.5 pH‐units wide for *X. bisporus* (FRR 0025), *A. penicillioides* (JH06THJ) and *E. halophilicum* (FRR 2471) respectively (Fig. 3). There was, however, a marked difference between strains. *X. bisporus* (FRR 0025) was unable to germinate at pH 8.8 but did so at a relatively high rate at pH 2.9, the lowest value tested; *A. penicillioides* (JH06THJ) did not germinate at pH 9.8 but did so at a reasonable rate at pH 3.0, the lowest value tested; and *E. halophilicum* (FRR 2471) germinated at a reasonable rates at pH 9.5 and 2.8, the most extreme values tested (Fig. 3).

**Figure 4:**
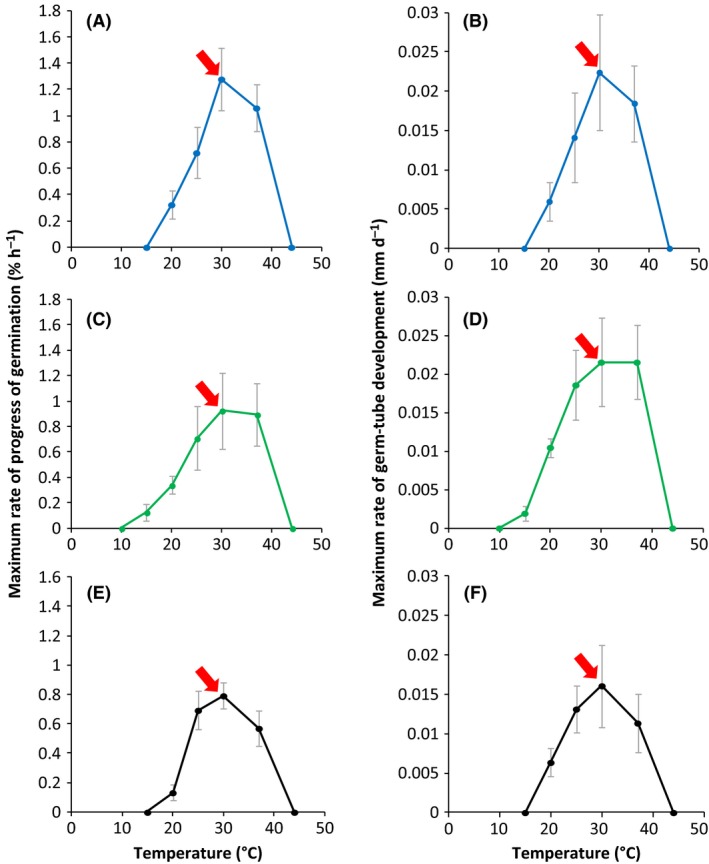
Maximum rates of spore germination (% of total h^−1^) and germ‐tube development for *Xeromyces bisporus* FRR 0025 (A and B), *Aspergillus penicillioides* JH06THJ (C and D) and *Eurotium halophilicum* (E and F) over a range of temperatures on malt‐extract, yeast‐extract phosphate agar (MYPiA) supplemented with diverse stressor(s) and incubated between 2 and 50°C (see Table 3). For *X. bisporus*, media were supplemented with glycerol (5.5 M)+sucrose (0.4 M); for *A. penicillioides* with glycerol (5.5 M)+ NaCl (1.2 M) and for *E. halophilicum* with glycerol (5.5 M)+NaCl (0.25 M)+sucrose (0.25 M). The red arrow indicates the temperature at which an additional study was carried out to assess the potency of glycerol as a determinant for the water‐activity limit for life (*Experimental procedures*; Stevenson *et al*., in press). Maximum rates of germination and germ‐tube development were determined from the curves (data not shown) and grey bars indicate standard errors.

The three temperature windows for germination of the xerophile strains were relatively similar, and spanned a range of approximately 30°C (Fig. 4). However, *A. penicillioides* appeared slightly more capable at low temperature; it was able to germinate at 15°C (Fig. 4C and D). The psychrotolerance of closely related strains was characterized in an earlier study which found that, in the presence of chaotropic substances (Ball and Hallsworth, 2015), mycelial growth occurred close to 0°C (Chin *et al*., 2010). The 30°C‐wide temperature windows for germination of the strains in the current study were not exceptionally wide by comparison with those of some bacterial strains (Santos *et al*., 2015).

### Optimum conditions for xerophilicity

For the three strains, optimum rates of germination and germ‐tube development were observed under similar conditions (Figs 1, 3 and 4). Generally, the optimum pH for germination lay between 4.5 and 7.7 (Fig. 3) and the optimum germination temperature was 30°C, although *A. penicillioides* (JH06THJ) was equally capable at 37°C (Fig. 4). The pH‐ and temperature optima are consistent with those reported for germination and mycelia growth of xerophiles (Gock *et al*., 2003; Williams and Hallsworth, 2009). However, rates of germination and germ‐tube development were slow for *X. bisporus* (FRR 0025) and *E. halophilicum* (FRR 2471) in pH‐ and temperature assays (Figs 3 and 4) because the water activity of germination media was sub‐optimal (i.e. in the range 0.729–0.717; Tables 2 and 3; Fig. 1). Germination and mycelial growth of xerophiles typically occur at the lowest water‐activity at ~30°C and pH values in the range 5.5–7.5 (Pitt, 1975; Gock *et al*., 2003; Williams and Hallsworth, 2009), and this is consistent with the high levels of xerophilicity observed in the current study at 30°C and in the pH range 6.30–7.20 (Table 1; Fig. 1).

### Asymmetrical response to *supra*‐ and sub‐optimal conditions

For sub‐ and *supra*‐optimal pH values, the decreases in germination and germ‐tube development were more or less equivalent (Fig. 3). By contrast, the germination curves for water activity and temperature are asymmetrical (Figs 1 and 4). Asymmetry can be observed in biological systems at various levels; from the chirality of metabolites (Neville, 1976; Clark, 1977) to asymmetrical stress mechanisms, growth kinetics or dynamics of ecosystem development (current study; McCammick *et al*., 2010; Cray *et al*., 2013a). In terms of cellular stress parameters, conditions which entropically disorder macromolecular systems are the most severely inhibitory; e.g. *supra*‐optimal water activities, temperatures or extreme chaotropicity (Figs 1 and 4; Hallsworth and Magan, 1994; Hallsworth *et al*., 1998, 2003b; Hallsworth *et al*., 2007; Bell *et al*., 2013; Cray *et al*., 2013a, 2015). By contrast, low temperature, low water‐activity and kosmotropic substances induce a more gradual decrease in microbial growth/metabolism (Figs 1 and 4; Chin *et al*., 2010).

There was considerable variation in germination and germ‐tube development within each spore population assayed, as indicated by error bars (Figs 1, 3 and 4). For fungal xerophiles, both intraspecies and intrastrain variability of spore phenotype/behaviour (see also Stevenson *et al*., in press) exemplify the natural variation inherent to biological systems (e.g. Hallsworth *et al*., 2003a; Cray *et al*., 2013a); a phenomenon which is even observed in populations of spores which have been harvested from an individual fungal colony. This is illustrated, for instance, by variation of survival which was demonstrated by the inactivation kinetics of fungal spores exposed to extreme stresses (Dijksterhuis *et al*., 2013). In addition, a population of spores has a distribution of lag‐phase times and rates of germination; both of these increase at low water‐activity. Dagnas *et al*. (2015) described an increase in the spread of germination times for conidia of *Penicillium corylophilum* treated with essential oils within red‐cabbage seed extract. The increase of variability within a cell population can act in such a way that enables the population to overcome/circumvent stress. For instance, heterogeneity of the germination process even occurs within the multicellular macroconidia of *Fusarium culmorum*, where germ tubes are formed on the apical cells in the majority of the cases (Chitarra *et al*., 2005). Interestingly, if apical cells were killed, the centrally located cells germinated giving the macroconidium a second chance (Chitarra *et al*., 2005). Such variation, observed in populations of diverse types of propagule, is an inherent part of the ecology of both plant ‐ and microbial species and may enhance the prevalence of a population within a specific habitat (Hill, 1977; Chitarra *et al*., 2005; Cray *et al*., 2013a; Oren and Hallsworth, 2014). Even the diversity of expressed genes can increase upon stress, as observed in *Aspergillus niger* conidia treated with the antifungal polyene natamycin (van Leeuwen *et al*., 2013). Phenotypic heterogeneity has also been reported for genetically homogeneous populations of bacteria (Touzain *et al*., 2010).

### Concluding remarks

Under extreme‐yet‐permissive water‐activity regimes, the germination of some of the most extremophilic microbes on Earth was characterized. Accordingly, the water activity windows for germination were extraordinarily wide (~1 to 0.651–0.637). The asymmetry of their germination kinetics reflected entropy‐level stress mechanisms which operate on cellular macromolecules. The biotic windows of these strains, kinetics of germination and germ‐tube development, symmetry/asymmetry of their stress phenotype and the inherent variation in behaviour within spore populations can act as determinants for ecological processes. Fundamental knowledge of xerophile behaviour based on this and other studies^1^ suggests that these strains may have potential as model systems which can be used to address scientific questions in the fields of extremophile biology, biosphere function, food spoilage, astrobiology, biophysics and synthetic biology. The current study gave rise to a number of unanswered questions: might glycerol be able to catalyse germination at the water‐activity limit for life (see *Experimental procedures*); under some conditions, can glycerol enable fungal germination at < 0.600 water activity; and can glycerol enhance the habitability of hostile environments. *A. penicillioides*, and other *Aspergillus* spp., are regularly found as contaminants of space craft, are highly tolerant to salt and low temperatures, and can function under microaerophilic or anaerobic conditions. It is pertinent, therefore to ask: what are the implications of abiotic glycerol, which has been identified in extraterrestrial locations, for potential contamination of other planetary bodies with terrestrial microbes during space‐exploration missions?

## Experimental procedures

### Fungal strains, media and culture conditions

*A. penicillioides* strain JH06THJ was isolated by Williams and Hallsworth (2009) and is available from the corresponding author of the current article. *E. halophilicum* 2 strain FRR 2471 and *X. bisporus* strain FRR 0025 were obtained from CSIRO Food and Nutritional Sciences Culture Collection (North Ryde, NSW, Australia). Cultures were maintained on malt‐extract, yeast‐extract phosphate agar (MYPiA; 10 g malt extract, 10 g yeast extract, 1 g anhydrous K_2_HPO_4_, agar 15 g l^−1^) supplemented with 5.5 M glycerol (0.821 water activity) at 30°C.

### Production of spores; germination assays

Spores were obtained from cultures incubated on MYPiA+glycerol (5.5 M) for 10–14 days for *A. penicillioides* and 21–28 days for *X. bisporus* and *E. halophilicum*. Spores were harvested from colonies growing on MYPiA+glycerol (5.5 M) media by covering Petri plates with sterile solutions of 5.5 M glycerol (15 ml); aerial spores were then dislodged by gently brushing with a sterile glass rod. The resulting suspension was passed through sterile glass‐wool twice, to remove hyphal fragments as described in earlier studies (Hallsworth and Magan, 1995; Chin *et al*., 2010). Spore suspensions were then adjusted to a final concentration of 1 × 10^6^ spores ml^−1^. Inoculation of germination media was carried out by pipetting the spore suspension (150 μl) onto the medium; the suspension was then distributed across the agar surface using a sterilize glass spreader.

Germination was assessed by removing a 4‐mm agar disc, and immediately quantifying percentage germination, spore diameter and germ‐tube length using a light microscope. Plates were immediately resealed and placed back in the incubator after removal of the agar discs. Percentage germination was determined via counts of 200 spores, and 50 individual germinated spores were measured for germ‐tube length; spores with germ‐tubes longer than their diameter were considered to have germinated (Hallsworth and Magan, 1995). In each case, percentage germination and mean germ‐tube length were determined for isolated spores and were not assessed for any spores located in clumps. Assessments were made at least daily over a 30‐day period.

The germination process was characterized over the entire windows of water activity, temperature and pH for these three xerophile strains. Water relations for germination were assessed on MYPiA‐based media supplemented, using a range of concentrations, with glycerol+sucrose for *X. bisporus* FRR 0025, glycerol+NaCl for *A. penicillioides* JH06THJ and glycerol+NaCl+sucrose *E. halophilicum* FRR 2471, as these media were highly permissive for germination of these strains in the low water‐activity range (Stevenson *et al*., in press). For the determination of germination rates over the pH range, media were supplemented with: glycerol (5.5 M)+sucrose (0.4 M) for *X. bisporus* FRR 0025, glycerol (5.5 M)+NaCl (1.2 M) for *A. penicillioides* JH06THJ and glycerol (5.5 M)+NaCl (0.25 M)+sucrose (0.25 M) for *E. halophilicum* FRR 2471 and buffered to give pH values of media from 2.90 to 9.80 (see Table 2). Germination was characterized over a temperature range using the following media: glycerol (5.5 M)+NaCl (1.2 M) for *A. penicillioides* JH06THJ, glycerol (5.5 M)+sucrose (0.4 M) for *X. bisporus* FRR 0025 and glycerol (5.5 M)+NaCl (0.25 M)+sucrose (0.25 M) for *E. halophilicum* FRR 2471 over the range 2–50°C (Table 3).

### Quantification of pH and water activity

The pH values for pre‐autoclaved media were determined using a Mettler Toledo Seven Easy pH‐probe (Mettler Toledo, Greifensee, Switzerland); values for solid media (post‐autoclaved) were determined prior to inoculation using Fisherbrand colour‐fixed pH indicator strips (Fisher Scientific Ltd, Leicestershire, UK). The water activity of all media was determined empirically using a Novasina Humidat‐IC‐II water‐activity machine fitted with an alcohol‐resistant humidity sensor and eVALC alcohol filter (Novasina, Pfäffikon, Switzerland). Water‐activity measurements were taken at the same temperature at which cultures were to be incubated and several precautions were employed to ensure accuracy of readings, as described previously (Hallsworth and Nomura, 1999; Stevenson *et al*., 2015a). The instrument was calibrated between each measurement using saturated salt solutions of known water activity (Winston and Bates, 1960). The water activity of each medium type was determined three times, and variation was within ± 0.001. Media chao‐/kosmotropicity values were determined using the agar‐gelation method described by Cray *et al*. (2013b). Extra‐pure reagent‐grade agar (Nacalai Tesque, Kyoto, Japan), at 1.5% w/v and supplemented with stressors at the concentrations used in the medium, was used to determine chao‐/kosmotropicity values for added solutes (see Hallsworth *et al*., 2003b; Cray *et al*., 2013b). A Cecil E2501 spectrophotometer fitted with a thermoelectrically controlled heating block was used to determine the wavelength and absorbance values at which to assay compounds, and values for chao‐/kosmotropic activity were calculated relative to those of the control (no added solute) as described by Cray *et al*. (2013b).

### Replication; analysis and presentation of data

All measurements were carried out in triplicate. The values for maximal rates of percentage germination for the three model strains over a range of water activity (0.995–0.640), pH (2.80–9.80) and temperature (10–44°C) (Figs 1, 3 and 4) were determined according to the exponential part of curves plotted for these parameters over time (data not shown). The data obtained are presented as the maximum rate of progress of germination (% of total spores h^−1^) versus water activity, pH and temperature (Figs 1, 3 and 4 respectively). Minimum water‐activity values at which germination occurred, within a 30‐day incubation period, were compared with those reported previously (Fig. 2). Optimum temperatures and pH values, and minimum water activity ranges, on which future studies to elucidate the potential role of glycerol as a determinant for the water‐activity limit for life should focus were identified and indicated (Figs 1, 3 and 4). A further study involving biophysically diverse types of culture media (see also Williams and Hallsworth, 2009; Stevenson *et al*., 2015a) was carried out to establish whether glycerol can enhance catalyse germination at the water‐activity limit for life (Stevenson *et al*., in press).

## Conflict of interest

None declared.

## Notes

1 Pitt (1975); Gock *et al*. (2003); Williams and Hallsworth (2009); Chin *et al*. (2010); Alves *et al*. (2015); Leong *et al*. (2015); Stevenson *et al*. (2015a); Cray *et al*. (2016).

2 Recently renamed as *Aspergillus halophilicus* (Hubka *et al*., 2013).
